# Emerging Therapies for Advanced Cholangiocarcinoma: An Updated Literature Review

**DOI:** 10.3390/jcm10214901

**Published:** 2021-10-24

**Authors:** Anthony Vignone, Francesca Biancaniello, Marco Casadio, Ludovica Pesci, Vincenzo Cardinale, Lorenzo Ridola, Domenico Alvaro

**Affiliations:** 1 Department of Translational and Precision Medicine, Sapienza University of Rome, Viale dell’Università 37, 00185 Rome, Italy; marco.casadio@uniroma1.it (M.C.); ludovica.pesci@uniroma.it (L.P.); lorenzo.ridola@uniroma1.it (L.R.); domenico.alvaro@uniroma1.it (D.A.); 2 Department of Medical-Surgical and Biotechnologies Sciences, Polo Pontino, Sapienza University of Rome, Corso della Repubblica 79, 04100 Latina, Italy; vincenzo.cardinale@uniroma1.it

**Keywords:** cholangiocarcinoma, targeted therapy, immunotherapy

## Abstract

Cholangiocarcinoma is a group of malignancies with poor prognosis. Treatments for the management of advanced-stage cholangiocarcinoma are limited, and the 5-year survival rate is estimated to be approximately 5–15%, considering all tumor stages. There is a significant unmet need for effective new treatment approaches. The present review is provided with the aim of summarizing the current evidence and future perspectives concerning new therapeutic strategies for cholangiocarcinoma. The role of targeted therapies and immunotherapies is currently investigational in cholangiocarcinoma. These therapeutic options might improve survival outcomes, as shown by the promising results of several clinical trials illustrated in the present review. The co-presence of driver mutations and markers of susceptibility to immunotherapy may lead to rational combination strategies and clinical trial development. A better understanding of immunologically based therapeutic weapons is needed, which will lead to a form of a precision medicine strategy capable of alleviating the clinical aggressiveness and to improve the prognosis of cholangiocarcinoma.

## 1. Introduction

Cholangiocarcinoma (CCA) is a rare malignant tumor that develops from the epithelium of the bile ducts or peribiliary glands (PBGs). Although CCA is considered a rare tumor in Western countries, it represents 3% of all gastrointestinal malignant tumors worldwide and the second most common primary liver cancer [1]. In Eastern countries, the incidence is higher than in Western ones, where it is estimated to be lower than 4 cases/100,000 people/year [2]. Northeast Thailand has the highest CCA rate in the world (90 cases/100,000 people/year) [3]. The highest incidence rate is in the seventh decade, with a slight prevalence in males. Due to classification coding (four different ICD-10 sub-codes) and variable terminology, CCA burden has been underestimated. CCA is the first cause of metastasis of unknown origin, and this further highlights how we still do not know the real burden of CCA [4]. While a reduction of the mortality rate from other cancers, including breast, lung, and colon cancer, has been observed in 1990–2009 (USA data), the mortality rate for liver and bile ducts tumors increased by more than 40% and 60% in females and males, respectively. While the mortality rate from hepatocellular carcinoma (HCC) has become more uniform across Europe, intrahepatic CCA mortality has substantially increased [5]. 

Anatomically, three types of cholangiocarcinoma can be distinguished: intrahepatic (iCCA), perihilar (pCCA) and distal (dCCA). Histologically, these are different kinds of tumors, considering cholangiocarcinogenesis as a process that starts from several cells of origin. In particular, pCCA and dCCA are mainly mucinous adenocarcinomas, while iCCA is highly heterogeneous, since it could resemble conventional mucinous adenocarcinomas (large-duct type iCCA), similar to p/dCCA, or transformed interlobular bile ducts (small-duct type iCCA).

Currently, surgical resection with negative margins represents the best potentially curative therapy of CCA. Therapeutic options for the management of advanced-stage CCA are limited, and the 5-year survival rate is estimated to be approximately 5–15%, considering all tumor stages [6]. Cisplatin plus gemcitabine (GEMCIS) represents the first-line treatment for these patients, as established by the phase II BT22 trial and the phase III ABC-02 trial [7,8].

Few studies have enrolled specifically iCCA patients or have reported the anatomic subtypes of CCA (iCCA, pCCA, and dCCA). Many studies reviewed here concerned biliary tract cancers (BTCs), enrolling together CCA and gallbladder cancer (GBC) patients. Neglecting CCA heterogeneity in the study design, in terms of anatomical, histological, and molecular subtypes, might represent a strong limitation in patients’ allocation to clinical trials. Moreover, given the possibilities shown by the development of targeted therapies, molecular profiling and efficient biomarkers would be needed to select the best therapeutic option for each patient [9].

The present review aims at summarizing the current evidence and future perspectives with regards to new therapeutic strategies for advanced CCA. Most drugs summarized in the following paragraphs are already used in the management of some oncological diseases, such as PD-L1 inhibitors (Pembrolizumab) that represent the first-line monotherapy for advanced non-small cell lung cancer (NSCLC) with a programmed death ligand 1 tumor proportion score of 50% or greater and without EGFR/ALK aberrations, based on the results of the phase III trial KEYNOTE 024 [10].

## 2. Targeted Therapy

### 2.1. FGFR2 Inhibitors

Approximately 15–20% of iCCAs have been observed to have FGRF2 translocations [11] (fusion or rearrangements), implicated in promoting cell proliferation and angiogenesis. These mutations are almost absent in extrahepatic cholangiocarcinomas. On this basis, several FGFR 1–3 inhibitors have been tested in advanced cholangiocarcinomas patients, showing good antitumor activity and safety. Particularly, the European Medicines Agency (EMA) approved in April 2021 the use of Pemigatinib for previously treated advanced cholangiocarcinomas showing FGFR2 fusion or rearrangement. Furthermore, a phase III study (FIGHT-302) [12] is currently ongoing to test the efficacy of Pemigatinib as a first-line treatment versus chemotherapy in patients with advanced cholangiocarcinoma with FGFR2 mutations (Table 1). The efficacy of Infigratinib (BGJ398), a reversible selective FGFR 1–3 inhibitor, is also under evaluation (NCT03773302) as a first-line treatment for patients with locally advanced or metastatic cholangiocarcinoma harboring FGFR2 mutations (Table 1).

However, point mutations of the FGFR 2 domain have been found capable of conferring resistance to FGFR inhibitors in previously treated patients [13]. In this category of patients, Futibatinib, a selective and irreversible FGFR inhibitor, has shown inhibitory activity and partial response, and a phase III study (Table 1) is underway to test its efficacy as a first-line treatment in patients with advanced CCA (FOENIX-CCA3 and NCT04093362). Another reversible ATP competitive inhibitor, Erdafitinib, showed promising result in a phase I–II study [14].

### 2.2. Metabolic Regulator (IDH Inhibitors)

Reprogramming of cancer cells’ metabolism has been defined as one of the hallmarks of cancer [15] and represents a possible target for precision medicine. Genomic and transcriptomic studies [16] have demonstrated that isocitrate dehydrogenase 1 and 2 (IDH1, IDH2) mutations occur in 13–25% of iCCA. These enzymes are involved in tricarboxylic acid cycle (TCA), β-oxidation of unsaturated fatty acids, response to oxidative stress, and expression of chromatin remodelers. In IDH1/2-mutated cells, the oncometabolite D-2-dihydroxyglutarate (2-HG) accumulates, leading to metabolic and epigenetic changes, enhanced proliferation, and susceptibility to DNA damage. This pathway may be hampered by inhibitors of IDH1 (AG120) and IDH2 (AG221), such as ivosidenib and enasidinib (NCT02273739), with encouraging results in randomized control trials (RCTs). Patients with IDH1-mutated iCCA who had progressed on previous therapy [17] showed a significant response to ivosidenib when compared to placebo-administered patients in the ClarIDHy phase III double-blind clinical trial (Table 1), in terms of both progression-free survival (2–7 vs. 1–4 months) and overall survival (10–8 vs. 9–7 months). Based on these results, ivosidenib has been recently approved by the FDA for locally advanced and metastatic cholangiocarcinoma with IDH1 mutations. IDH1 inhibitors are currently under investigation also in combination with other treatments. A phase Ib/II basket trial is evaluating Olutasidenib (FT-2102) alone, in combination with azacitidine, nivolumab, or gemcitabine and cisplatin in 200 patients with different solid tumors harboring the same IDH1 mutations (NCT03684811).

### 2.3. Tyrosine Kinase Inhibitors

Mutations of epidermal growth factor receptors play a pivotal role in different cancers [18], and several drugs are already approved for specific subsets of malignancies, i.e., EGFR-mutated non-small cell lung cancer [19] and colorectal cancer [20]. Nevertheless, convincing evidence of their efficacy in CCA is still lacking.

In the PiCCA phase II randomized clinical trial [21], panitumumab, a monoclonal anti-EGFR1 antibody, was administered in combination with gemcitabine and cisplatin in KRAS-wild-type patients versus gemcitabine and cisplatin alone, but it failed to improve ORR, PFS, and OS. Similar results were obtained in a phase II study in chemotherapy-naive patients with advanced BTC, treated with panitumumab and GEMOX and GEMOX alone. Despite the attempt of selecting patients by IHC, PCR, and Sanger sequencing for KRAS, BRAF, and PI3KCA, no significant survival differences were observed. Nevertheless, it needs to be underlined that the cohorts of these two studies were not specifically tested for enrichment in EGFR alterations [22]. In addition, a phase II clinical trial studied the efficacy of cetuximab combined with GEMOX vs. GEMOX alone in advanced BTC patients; KRAS, NRAS, and BRAF mutations and EGFR expression, were the criteria selected to stratify these patients. Despite a significant difference in progression-free survival, the study did not reach the primary endpoint (ORR) nor demonstrated a higher OS in the cetuximab arm. However, other genetic alterations involved in the EGFR pathway, i.e., ROS1, ALK, or c-MET [23], were not specifically investigated and might have a role in explaining anti-EGFR resistance.

The EGFR inhibitor erlotinib (Table 1) was studied in combination with chemotherapy regimens [24] and bevacizumab [25], but no clear survival benefits were observed when compared to current standard of care. Varlitinib, a competitive inhibitor of the tyrosine kinases EGRF and HER 2–4, is currently under investigation in monotherapy (phase II, NCT02609958) and in combination with capecitabine in advanced BTC patients (phase II/III, NCT03093870) (Table 1).

As far as the HER family is concerned, molecular profiling studies [26] have underlined the frequency of ERRB2 aberrations in p/dCCA, but evidence about the efficacy of anti-HER2 drugs in CCA has not supported their use in clinical practice so far [1]. On these bases, the feasibility of this treatment has already been demonstrated [27], and several phase II clinical trials are currently evaluating the efficacy of combination treatments with trastuzumab and tucatinib (NCT04579380) and with chemotherapy (NCT04430738).

Combination treatments with bevacizumab and gemcitabine or capecitabine have been tested in a multicenter phase II trial, given the high prevalence of VEGF overexpression in CCA [28]. Nevertheless, the patients were not selected based on their mutational profile, and this may be responsible for the poor outcome of the study. 

The lack of patients’ stratification may have also affected the results of different clinical trials that evaluated the multikinase inhibitor sorafenib, also targeting VEGFR2 and 3 [29]. Adding sorafenib to GEM–CIS in biliary tract cancer showed increased treatment toxicity without simultaneous clinical benefits in a phase II RCT [30] including biliary adenocarcinomas of all subtypes without taking into account histological and molecular differences. Sun et al. [31] have shown that regorafenib improved PFS of (15.6 weeks) and OS (31.8 weeks) in advanced BTC patients with disease progression after first-line therapy. Targeting neurotrophic tyrosine kinase receptor (NTKR) fusions has seemed promising, too [32]. Two phase II basket trials have investigated entrectinib [33] and larotrectinib [34]. FDA and EMA have approved larotrectinib and entrectinib as “wildcard” drugs that can be used in every kind of malignancy harboring this genetic alteration, regardless of the anatomical origin. Unfortunately, NTKR fusions are rarely detected in CCA [35].

### 2.4. Proteasome Inhibitors

Mutations/deletions of the PTEN gene were observed in approximately 5% of iCCAs associated with poor prognosis [6]. It was also observed that PTEN mutation/deletion is also associated with increased activity of proteasomes in iCCAs. On these bases, a phase III study (Table 1) is actually evaluating the efficacy of Bortezomib, a proteasome inhibitor, in patients with advanced iCCA who have progressed after at least two cycles of systemic chemotherapy (NCT03345303).

## 3. Immunotherapy

Since 2010, immunotherapy has been one of the most important strategies in the treatment of malignancies, together with surgery, chemotherapy, radiotherapy, and targeted therapy, even if its efficacy is very variable, and only a percentage of patients obtain a durable response [36]. The mechanism of immunotherapy is to enhance the anti-tumor immune response, including both adaptative cells (B and T cells) and innate cells such as macrophages, neutrophils, natural killers. Immunotherapy includes immune checkpoint inhibitors (ICIs) targeting programmed death 1 (PD-1), programmed death-ligand 1 (PD-L1), and cytotoxic T lymphocyte antigen-4 (CTLA-4), cancer vaccines, and adoptive cell transfer (ACT). Several factors can influence the effect of immunotherapy-based treatments: the environment of tumor and immune cells, vascularization, extracellular matrix, and molecular signaling pathway [37]. Several therapeutic options in patients affected by biliary tract cancers are under investigation, such as immunotherapeutic strategies with checkpoint inhibitors, peptide- and dendritic cell-based vaccines, and adoptive cell therapy, in monotherapy or in combination with targeted therapy and/or chemotherapy. Nowadays, scientific evidence on the use of immunotherapy in CCA are limited, although different trials are currently investigating the role of anti-CTLA-4 monoclonal antibodies, the targeting of PD-L1 or its receptor, PD-1, and chimeric antigen receptor T (CAR-T) cell immunotherapy. Unfortunately, checkpoint inhibitor monotherapy has shown low efficacy in CCA patients. Indeed, Pembrolizumab, a PD-L1 inhibitor, demonstrated a median progression-free survival of 1.8 months in patients affected by CCA in the phase Ib basket trial KEYNOTE 028 [38]. Checkpoint inhibitors showed encouraging results in patients with microsatellite instability or DNA mismatch repair in the KEYNOTE 158 trial [39], even if only a small percentage of patients with a positive response to this kind of treatment reported a better clinical response [40]. Pembrolizumab demonstrated good efficacy in a recent Korean study that retrospectively analyzed 51 patients with PD-L1-positive CisGem-refractory biliary tract cancer. In PD-L1-positive patients, pembrolizumab showed durable efficacy, with a 9.8% response rate with manageable adverse events. Ongoing studies and clinical trials are currently exploring combined immunotherapeutic approaches targeting both the innate and the adaptive immune system, and/or combined strategies also involving chemotherapy or radiation.

Particularly, there are many ongoing phase I–III trials exploring the role of targeting PD-L1, its receptor PD-1, anti CTL-A4 with monoclonal antibodies in monotherapy or in combination with chemotherapy, targeted therapy, local ablative therapy, and the role of CAR-T cell immunotherapy in biliary tract cancer (Table 2 and Table 3). In particular, KEYNOTE-028 and KEYNOTE-158, two multicentric, non-randomized, open-label, phase IB and II trials, showed a durable antitumor activity of Pembrolizumab in 6–13% of patients with advanced BTC. In KEYNOTE-158, they observed a median progression free survival (PFS) of 2.0 months and a Median overall survival (OS) of 7.4 months; adverse events were mainly mild to moderate in severity [39]. Another immunotherapeutic agent, Nivolumab showed a response rate of 22% and a disease control rate of 59% in a Phase II multi-institutional study including 46 patients affected by advanced biliary tract cancer in second-line therapy [41].

The combination of immunotherapy and chemotherapy looks promising. Two Phase III trials are evaluating the efficacy and safety of KN035 plus Gemcitabine–Oxaliplatin compared to standard of care Gemcitabine–Oxaliplatin therapy (NCT03478488) and the association of Durvalumab and Gemcitabine plus cisplatin (NCT03875235). BilT-01, a multicenter randomized Phase II trial, described a prolonged PFS six months after the addition of nivolumab to gemcitabine and cisplatin (NCT02829918) [42]. LEAP 005 demonstrated a promising antitumor activity and manageable toxicity of Pembrolizumab in combination with Lenvatinib in 31 patients affected by BTC [43]. 

Regarding Adoptive Cell Therapy (ACT), a phase III, non-randomized trial is studying the role of cytokine-induced killer cells in association with radiofrequency ablation in 50 patients with CCA (NCT02482454).

## 4. Clinical-Pathological and Radiomic Monotherapy Susceptibility in Patients with Cholangiocarcinoma

Within the CCA clinical-pathological spectrum, the pattern of tumor growth has been correlated with specific histological features, e.g., small-bile duct iCCAs and cholangiolocarcinoma (CLC) showed a mass-forming growth pattern, while large-bile duct iCCAs showed both a mass-forming growth pattern and a combination of a mass-forming growth pattern with a periductal infiltrative growth pattern, the latter being the typical pattern of growth of pCCA [44]. Mass-forming iCCAs showed more heterogeneous clinical-pathological characteristics than other gross types [45]. Radiologically, at dynamic contrast-enhanced imaging, all large-bile duct iCCAs showed concentric filling at the venous phase, whereas small-bile duct iCCAs/CLCs showed washout in various patterns, in a clinical-pathological study including correlates with magnetic resonance imaging [44].

The USA Food and Drug Administration approved the use of pembrolizumab for patients with advanced solid tumors lacking the expression of mismatch repair (MMR) proteins (MLH1, MSH2, MSH6, and PMS2) or having high microsatellite instability (MSI-H) [46]. MMR proteins can be inactivated through somatic or germline mutations or they can be silenced through promoter hypermethylation, e.g., of the MLH1 gene [47]. These alterations culminate to hypermutation during DNA replication (MSI) and may lead to the development of malignancies [48]. Interestingly, such molecular alterations predispose to an increase of the neoantigen load of the tumor, promoting susceptibility to immunotherapies targeting the PD-1 pathway because of the increased inflammation surrounding these tumors [40].

Given the potential for immunotherapy in patients with CCA, authors studied the expression of PD-L1/PD-1 and evaluated the presence of associated genetic alterations. For example, in 652 biliary tract cancers that comprised 77 p/dCCA, 372 iCCA, and 203 gallbladder cancer (GBC), 8.6% tumors were PD-L1-positive [GBC 12.3% (25/203), iCCA 7.3% (27/372), and p/dCCA 5.2% (4/77)]. Interestingly, there was an increase in BRAF, BRCA2, RNF43, and TP53 mutations in the PD-L1-positive group with respect to the PD-L1-negative one. Furthermore, there was an association between PD-L1 expression and certain biomarkers (TOP2A, TMB high, MSI-H). As noted by the authors, the aforementioned combinations of molecular alterations might direct the use of rational combination strategies and clinical trial development [49]. On the same line, Ju et al. analyzed 96 cases of CCA for morphology using H&E staining and for mutations of MMR genes using immunohistochemical staining. The authors found that 6% of the samples showed MMR deficiency (MMR-d). Divided by location, 10% (3 of 31) of iCCA and 5% (3 of 65) of p/dCCA were MMR-d. The best predictive factor for MMR-d was a nontypical infiltrating pattern of invasion [50].

The increasing awareness of CCA heterogeneity at the morphological and molecular levels, together with the advent of radiomic, artificial intelligence (AI), and machine learning, has revitalized the study of radiological correlates. For example, it has been shown that the magnetic resonance imaging texture signature, including three wavelets and one 3D feature, has the ability to discriminate inflamed from non-inflamed immunophenotypes based on the density of CD8+ T cells. This may be a surrogate of the response to immune checkpoint blockade [51]. The preoperative prediction of PD-1/PD-L1 expression and outcome in iCCA patients using magnetic resonance biomarkers and a machine learning approach has been attempted [52]. Utilizing qualitative and quantitative imaging traits, reasonable accuracy in predicting tumor grade and higher AJCC stage in iCCA has been shown [53].

## 5. Conclusions

The role of targeted therapy and immunotherapy in the treatment of CCA is currently under investigation. These options might improve survival outcomes (OS and PFS), as shown by the promising results of several clinical trials illustrated in the present review. This is even more important considering the poor therapeutic options in the management of CCA. The co-presence of driver mutations and markers of susceptibility to immunotherapy may lead to rational therapeutic combination strategies and clinical trial development. The combination of new therapeutic strategies, such as targeted therapy and immunotherapy, with conventional chemotherapy and/or locoregional treatments could be the next frontier for the treatment of advanced CCA. The evaluation of innovative strategies for the prediction of immunotherapy susceptibility, such as multi omics, preferably within longitudinal clinical trials, and the use of systems of data analysis based on the precepts of AI, may circumvent the lack of therapeutic biomarkers for immunotherapy. A better understanding of immunological-based therapeutic weapons is needed, which will lead to a form of a precision medicine strategy capable of alleviating the clinical aggressiveness and to improve the prognosis of CCA.

## Figures and Tables

**Table 1:** Phase III targeted-therapy trials for BTC.

NCT	Phase	Condition or Disease	N. Patients	Regimen	Line of Therapy	Results
NCT02989857 ClarIDHy	III	Advanced and Metastatic CCA	187	Ivosidenib	II	OS: 8–10 months Median PFS: 2–7 months
NCT01149122	III	Advanced BTC	103	GEMOX + Erlotinib	I	ORR: 48% Median PFS: 7.3 months OS: 10.7 months
NCT03093870	II/III	BTC	151	Varlitinib + Capecitabine	I	ORR: 9.4% Median PFS: 2.8 months
NCT03345303	III	iCCA	50	Bortezomib	II	-
NCT03656536 Fight302	III	Advanced, CCA	432	Pemigatinib	I	ORR: 35.5% Median PFS: 6.93 months
NCT03773302	III	Advanced CCA	384	Infigratinib	I	-
NCT04093362	III	Advanced CCA	216	Futibatinib	I	-

**Table 2:** Ongoing immunotherapy trials of biliary tract cancers.

NCT	Phase	Condition or Disease	Number of Patients	Regimen	Status
ICI MONOTHERAPY					
NCT03110328	II	Advanced or refractory BTC	33	Pemrolizumab	Recruiting
NCT02054806 KEYNOTE-28	IB	Incurable advanced PD-L1 positive cancers, including BTC	477	Pembrolizumab	Completed
NCT02628067 KEYNOTE-158	IIA	Advanced, refractory solid cancer including BTC	1595	Pemrolizumab	Recruiting
NCT02829918	II	Advanced refractory BTC	54	Nivolumab	Active, not recruiting
NCT03867370	IB-II	Operable HCC o iCC	40	Toripalimab	Recruiting
DUAL ICI					
NCT03101566	II	BTC	75	Nivolumab+ Ipilimumab	Active, not recruiting
ICI IN COMBINATION WITH CHEMOTHERAPY					
NCT03473574	II	Naïve BTC	128	Durvalumab + tremelimumab + GEM or GEMCIS vs. GEMCIS chemotherapy	Active, not recruiting
NCT03046862	II	Unresectable, untreated BTC	31	Durvalumab + Tremelimumab + GEMCIS chemotherapy	Recruiting
NCT03704480	II	Advanced BTC	106	Durvalumab + tremelimumab + paclitaxel	Recruiting
NCT03875235	III	Advanced BTC	757	Durvalumab + GEMCIS vs GEMCIS + chemotherapy	Recruiting
NCT03257761	Ib	Unresecable, refractory HCC, PDAC, BTC excluding ampullary	90	Durvalumab + guadecitabine	Recruiting
NCT03111732	II	Unresecable, refractory BTC	11	Pemrolizumab + Oxaliplatine + Capecitabine	Active, not recruiting
NCT03260712	II	Unresecable, untreated BTC	50	Pemrolizumab + GEMCIS	Recruiting
NCT03796429	II	Advanced BTC	40	Gemcitabine + Toripalimab	Recruiting
NCT03101566	II	Unresecable, untreatable BTC	75	Nivolumab + Ipilimumab vs GEMCIS + Nivolumab	Active, not recruiting
NCT03785873	I/II	Unresecable, refractory BTC	40	Nivolumab + nal-irinotecan + 5-fluorouracil + leucovorin	Recruiting
NCT03478488	III	Unresecable, untreatable BTC	480	KN035 + GEMOX vs. GEMOX + chemotherapy	Recruiting
ICI IN COMBINATION WITH TARGETED THERAPY					
NCT03797326	II	Advanced, refractory solid tumours, including BTC	590	Lenvatinib + pembrolizumab	Recruiting
NCT02393248	I/II	Advanced solid tumour malignancy, including CCA		Pembrolizumab +pemigatinib	Recruiting
NCT03684811	I/II	BTC, iCC and other Hepatobiliary Carcinomas with IDH1 mutation	200	Nivolumab +FT-2102	Active, not recruiting
NCT03201458	Phase II	Metastatic BTC or gallbladder cancer	76	Atezolizumab + Cobimetinib	Active, not recruiting
NCT03639935	Phase II	Advance metastatic BTC	35	Nivolumab + Rucaparib	Recruiting
NCT03991832	Phase II	Solid tumours including IDH-mutated CCA	78	Olaparib and Durvalumab	Recruiting
ICI IN COMBINATION WITH LOCAL ABLATIVE THERAPY					
NCT02821754	II	Refractory or unresecable HCC or BTC	90	Durvalumab + Tremelimumab, Durvalumab + Tremelimumab + procedure (RFA or TACE or Cryoablation)	Recruiting
NCT03898895	II	Unresecable iCCA, eligible for RT	184	Pembrolizumab + SBRT	Recruiting
NCT03482102	II	Unresecable HCC or BTC	70	Durvalumab + tremelimumab + RT	Recruiting
TME TARGETED THERAPY					
NCT03314935	I/II	Malignant tumours including BTC	149	INCB001158 + FOLFOX/gemcitabine + cisplatin/paclitaxel	Active, not recruiting
NCT03329950	I	Malignant tumours including CCA	260	CDX-1140 (CD40 antibody), either alone or in combination with CDX-301 (FLT3L), pembrolizumab, or chemotherapy	Recruiting
NCT03071757	I	Locally advanced or metastatic solid tumours including CCA	170	ABBV-368 and ABBV-368 + Budigalimab (ABBV-181)	Active, not recruiting
ACT THERAPY					
NCT03820310	II	iCC after radical resection	20	Autologous Tcm Cellular Immunotherapy Combined with Traditional Therapy	Recruiting
NCT03801083	II	Locally Advanced, Recurrent, or Metastatic BTC	59	Tumour Infiltrating Lymphocytes	Recruiting
NCT03633773	I/II	iCC	9	MUC-1 CAR-T cell immunotherapy after fludarabine and cyclophosphamide	Recruiting
NCT02482454	III	Unresected CCA, withoutextrahepatic metastasis	50	Autologous cytokine-induced killer cells (CIK) after RFA	Active, not recruiting

ACT: adoptive cellular therapy, BTC: biliary tract cancer, CAR-T cell: chimeric antigen receptor T cell, CCA: cholangiocarcinoma, FOLFOX: folinic acid (leucovorin) + 5-fluorouracil + oxaliplatin, GEM: gemcitabine, GEMCIS: gemcitabine + cisplatin, HCC: hepatocellular carcinoma, iCC: intrahepatic cholangiocarcinoma, ICI: immune-checkpoint inhibitors, MUC-1: mucin 1, PDAC: pancreatic ductal adenocarcinoma, RFA: radiofrequency ablation, RT: radiotherapy, SBRT: stereotactic body radiation therapy, TACE: trans-arterial chemo embolization, TME: tumor microenvironment.

**Table 3:** Ongoing immunotherapy trials for BTC with preliminary results.

NCT	Phase	Condition or Disease	N. Patients	Regimen	Results
NCT02054806 KEYNOTE-28	IB	Incurable advanced PD-L1 positive cancers, including BTC	477	Pembrolizumab	ORR: 13% Median PFS: 2 months
NCT02628067 KEYNOTE-158	IIA	Advanced, refractory solid cancer including BTC	1595	Pemrolizumab	ORR: 5.8% Median PFS: 1.8 months
NCT02829918	II	Advanced refractory BTC	54	Nivolumab	ORR: 22% Median PFS: 3.8 monthd
NCT03797326	II	Advanced, refractory solid tumours, including BTC	590	Lenvatinib + pembrolizumab	ORR: 16%
